# Producing fast and active Rubisco in tobacco to enhance photosynthesis

**DOI:** 10.1093/plcell/koac348

**Published:** 2022-12-06

**Authors:** Taiyu Chen, Saba Riaz, Philip Davey, Ziyu Zhao, Yaqi Sun, Gregory F Dykes, Fei Zhou, James Hartwell, Tracy Lawson, Peter J Nixon, Yongjun Lin, Lu-Ning Liu

**Affiliations:** National Key Laboratory of Crop Genetic Improvement and National Center of Plant Gene Research, Huazhong Agricultural University, Wuhan 430070, China; Institute of Systems, Molecular and Integrative Biology, University of Liverpool, Liverpool L69 7ZB, UK; Department of Life Sciences, Sir Ernst Chain Building-Wolfson Laboratories, Imperial College London, South Kensington Campus, London SW7 2AZ, UK; School of Life Sciences, University of Essex, Colchester CO4 4SQ, UK; Department of Life Sciences, Sir Ernst Chain Building-Wolfson Laboratories, Imperial College London, South Kensington Campus, London SW7 2AZ, UK; Institute of Systems, Molecular and Integrative Biology, University of Liverpool, Liverpool L69 7ZB, UK; Institute of Systems, Molecular and Integrative Biology, University of Liverpool, Liverpool L69 7ZB, UK; National Key Laboratory of Crop Genetic Improvement and National Center of Plant Gene Research, Huazhong Agricultural University, Wuhan 430070, China; Institute of Systems, Molecular and Integrative Biology, University of Liverpool, Liverpool L69 7ZB, UK; School of Life Sciences, University of Essex, Colchester CO4 4SQ, UK; Department of Life Sciences, Sir Ernst Chain Building-Wolfson Laboratories, Imperial College London, South Kensington Campus, London SW7 2AZ, UK; National Key Laboratory of Crop Genetic Improvement and National Center of Plant Gene Research, Huazhong Agricultural University, Wuhan 430070, China; Institute of Systems, Molecular and Integrative Biology, University of Liverpool, Liverpool L69 7ZB, UK; College of Marine Life Sciences, and Frontiers Science Center for Deep Ocean Multispheres and Earth System, Ocean University of China, Qingdao 266003, China

## Abstract

Ribulose-1,5-bisphosphate carboxylase/oxygenase (Rubisco) performs most of the carbon fixation on Earth. However, plant Rubisco is an intrinsically inefficient enzyme given its low carboxylation rate, representing a major limitation to photosynthesis. Replacing endogenous plant Rubisco with a faster Rubisco is anticipated to enhance crop photosynthesis and productivity. However, the requirement of chaperones for Rubisco expression and assembly has obstructed the efficient production of functional foreign Rubisco in chloroplasts. Here, we report the engineering of a Form 1A Rubisco from the proteobacterium *Halothiobacillus neapolitanu*s in *Escherichia coli* and tobacco (*Nicotiana tabacum*) chloroplasts without any cognate chaperones. The native tobacco gene encoding Rubisco large subunit was genetically replaced with *H. neapolitanu*s Rubisco (*Hn*Rubisco) large and small subunit genes. We show that *Hn*Rubisco subunits can form functional L_8_S_8_ hexadecamers in tobacco chloroplasts at high efficiency, accounting for ∼40% of the wild-type tobacco Rubisco content. The chloroplast-expressed *Hn*Rubisco displayed a ∼2-fold greater carboxylation rate and supported a similar autotrophic growth rate of transgenic plants to that of wild-type in air supplemented with 1% CO_2_. This study represents a step toward the engineering of a fast and highly active Rubisco in chloroplasts to improve crop photosynthesis and growth.

## Teaser

Introducing a proteobacterial Rubisco with a greater carboxylation rate and a higher content of active sites into tobacco chloroplasts supports photosynthesis and growth at high CO2 concentrations.

**IN A NUTSHELL**

**Background:** Rubisco is the key enzyme responsible for fixing CO_2_. However, due to its intrinsically low catalytic turnover rate, Rubisco represents the ultimate rate-limiting step in plant photosynthesis. Improving Rubisco carboxylation and assembly in plants has been a long-standing challenge in crop engineering to meet the pressing need for increased global food production. There is mounting interest in replacing endogenous plant Rubisco with active non-native Rubisco candidates from other organisms to enhance photosynthetic carbon fixation.

**Question:** The folding and assembly of Rubisco in chloroplasts are intricate processes that usually require a series of ancillary factors. Seeking a new Rubisco variant that can be produced in chloroplasts with a high yield and high catalytic performance, without the requirement for cognate assembly factors and activases, could help improve carbon fixation in crop plants.

**Finding:** In this work, we introduced a Rubisco from a proteobacterium into tobacco chloroplasts to replace native tobacco Rubisco. In the proteobacteria, Rubisco is naturally encapsulated at a high density within a CO_2_-fixing protein organelle, the carboxysome. The foreign Rubisco derived from bacteria formed efficiently and was functional in chloroplasts without the need for exogenous chaperones. Intriguingly, the chloroplast-expressed bacterial Rubisco supported the autotrophic growth of transgenic plants at a similar rate to wild-type plants at 1% CO_2_.

**Next Step:** The successful production of functional bacterial Rubisco represents a step toward installing faster, highly active Rubisco, functional carboxysomes, and eventually active CO_2_ concentration mechanisms into chloroplasts to improve Rubisco carboxylation, with the intent of enhancing crop photosynthesis and crop yield on a global scale.

## Introduction

To meet the rising demands for food, an estimated 60%–110% increase in global agricultural production is strategically required by 2050 (Tilman et al., 2011; Price et al., 2013; Ray et al., 2013). However, the current trajectory for crop yields per unit area of land is apparently inadequate to nourish the increasing global population (Long et al., 2015; Kromdijk et al., 2016). Meanwhile, agriculture and related land-use changes generate roughly one-quarter of global CO_2_ emissions. It is thus imperative to develop new biotechnological strategies for enhancing plant photosynthesis, sustainable crop production, and resilience in a changing climate (Bailey-Serres et al., 2019).

Ribulose-1,5-bisphosphate carboxylase/oxygenase (Rubisco) is the essential enzyme responsible for carbon fixation in plants and is the most abundant protein on Earth (Bar-On and Milo, 2019; Sui et al., 2020; Liu, 2022). Rubisco catalyzes the incorporation of inorganic CO_2_ to produce a sugar precursor through the Calvin–Benson–Bassham cycle. Among the distinct evolutionary lineages of Rubisco found in nature (Bracher et al., 2017), Form I Rubisco has been the focus of most fundamental and engineering studies. Form I Rubisco is an L_8_S_8_ hexadecamer consisting of eight large subunits (L, ∼50 kDa) and eight small subunits (S, ∼15 kDa). Based on sequence homology, Form I Rubisco can be further phylogenetically subdivided into four distinct classes: A, B, C, and D (Tabita, 1999). Plants, β-cyanobacteria, and green algae contain the prevalent Form IB Rubisco, whereas marine α-cyanobacteria and some proteobacteria possess Form IA Rubisco (Shih et al., 2016). Form IA and Form IB Rubisco have different evolutionary ancestors and differ in protein sequence and electrostatic surface properties (Nakai et al., 2012; Zarzycki et al., 2013; Shih et al., 2016).

Despite its high productivity on a global scale, Rubisco is surprisingly inefficient, making the catalytic reactions of Rubisco the limiting step in photosynthetic CO_2_ fixation. The ineffectiveness of Rubisco originates from its slow carboxylation rate and restricted capability in discriminating between CO_2_ and O_2_. The oxygenation reaction of Rubisco, using O_2_ as a substrate, leads to photorespiration and causes a significant loss of photosynthetic production (Bauwe et al., 2010; Bracher et al., 2017; Flamholz et al., 2019). To overcome the inherent limitations of Rubisco, C_4_ and crassulacean acid metabolism (CAM) plants, algae, cyanobacteria, as well as some chemoautotrophs have evolved various forms of CO_2_-concentrating mechanisms (CCMs) to accumulate CO_2_ around Rubisco for enhancing carboxylation and suppressing oxygenation (Hennacy and Jonikas, 2020). By contrast, an overwhelming majority of agricultural crops, namely C_3_ plants, lack any form of CCM (Price et al., 2013); they produce Rubisco with relatively high CO_2_-binding affinities but low carboxylation rates. To ensure efficient carbon fixation, C_3_ plants produce higher levels of Rubisco (up to 30% of the total leaf nitrogen) than other species containing CCM (for example, 5%–9% of the total leaf nitrogen in C_4_ plants) (Feller et al., 2008). Engineering Rubisco with improved catalytic properties and introducing functional CCM into crop plants have been promising targets for improving photosynthesis and plant growth and increasing nitrogen use efficiency (Parry et al., 2013; McGrath and Long, 2014; Gonzalez-Esquer et al., 2016; Long et al., 2016; Rae et al., 2017; Sharwood, 2017; Iñiguez et al., 2021).

Despite recent advances in plastid transformation technology (Bock, 2015; Ruf et al., 2019), improving Rubisco kinetics and assembly in transplastomic plants has been a long-standing challenge in crop engineering (Erb and Zarzycki, 2016). Efforts have been made to identify new Rubisco variants with higher turnover rates from diverse natural species or hybrid Rubisco to replace endogenous plant Rubisco (Sharwood et al., 2016b; Conlan and Whitney, 2018; Flamholz et al., 2019; Davidi et al., 2020; Matsumura et al., 2020). Mathematical modeling suggested that introducing Rubisco with a high carboxylation rate could potentially lead to an over 25% increase in crop yields (Zhu et al., 2004). However, the challenges of engineering a non-native Rubisco into plants include inefficient assembly and poor solubility of heterologously expressed Rubisco (Wilson and Hayer-Hartl, 2018).

Although Rubisco variants can be expressed and assembled to form functional complexes in *Escherichia coli* (Davidi et al., 2020), co-expression of ancillary factors is in many cases necessary for the efficient folding and assembly of foreign Rubisco in transgenic chloroplasts (Whitney et al., 2015; Aigner et al., 2017; Wilson and Hayer-Hartl, 2018; Hayer-Hartl and Hartl, 2020). The assembly of functional Form IB Rubisco in plants requires cognate chaperones that are likely species specific; for example, up to seven cognate chaperones are involved in Rubisco assembly in *Arabidopsis thaliana* (Aigner et al., 2017). Moreover, the large and small subunits of plant Rubisco are encoded in disparate locations: the plant Rubisco large subunit RbcL is encoded by a single *rbcL* gene in the chloroplast genome, whereas the small subunit RbcS, which plays a vital role in regulating Rubisco content (Mao et al., 2022), is encoded by multiple *rbcS* genes in the nuclear genome. All these factors unambiguously increase the complexity of engineering and modifying Rubisco in plants (Whitney et al., 2011a; Martin-Avila et al., 2020) and may contribute to the observed lower yields of exogenous Rubisco in transgenic lines (∼10% of the Rubisco content of the wild-type [WT]) (Lin et al., 2014; Long et al., 2018; Orr et al., 2020). A “red-type” Form IC Rubisco was recently expressed in transplastomic tobacco (*Nicotiana tabacum*) with ∼30% of the Rubisco content of the WT, but the Rubisco activity relied strictly on co-expression of the cognate CbbX activase (Gunn et al., 2020). To date, transplastomic plants expressing non-native Rubisco required high levels of CO_2_ for proper growth, and growth performance was worse than that of the WT, even under high CO_2_ concentrations (Lin et al., 2014; Long et al., 2018; Wilson et al., 2018; Gunn et al., 2020; Orr et al., 2020). It is thus desirable to identify suitable Rubisco candidates that can be functionally assembled in chloroplasts without the requirement for cognate assembly factors and activases.

The chemoautotrophic bacterium *Halothiobacillus* (*H*.) *neapolitanus* contains Form 1A Rubisco, which is encapsulated at a high density in α-carboxysomes (Sun et al., 2022). Previous studies have demonstrated that functional *H. neapolitanus* α-carboxysomes containing Rubisco can be heterologously formed without assembly chaperones in *E. coli* (Bonacci et al., 2012; Baumgart et al., 2017; Chen et al., 2022), providing a promising Rubisco with minimal assembly requirement for plant engineering. Here, we expressed *H. neapolitanus* Rubisco large and small subunits CbbL and CbbS in both *E. coli* and tobacco chloroplasts without exogenous assembly factors, resulting in a high yield of functional Form 1A Rubisco CbbL_8_S_8_ complexes (∼40% of the Rubisco content of the WT). We demonstrate that the engineered Rubisco has a greater carboxylation rate and can support essentially the same growth of transgenic lines as that of WT tobacco in air supplemented with 1% CO_2_. Our study provides insight into the diversity of Rubisco assembly and offers promising strategies for Rubisco bioengineering to enhance photosynthetic performance and crop growth.

## Results

### Reconstitution of *Hn*Rubisco in *E. coli*

**Figure 1:**
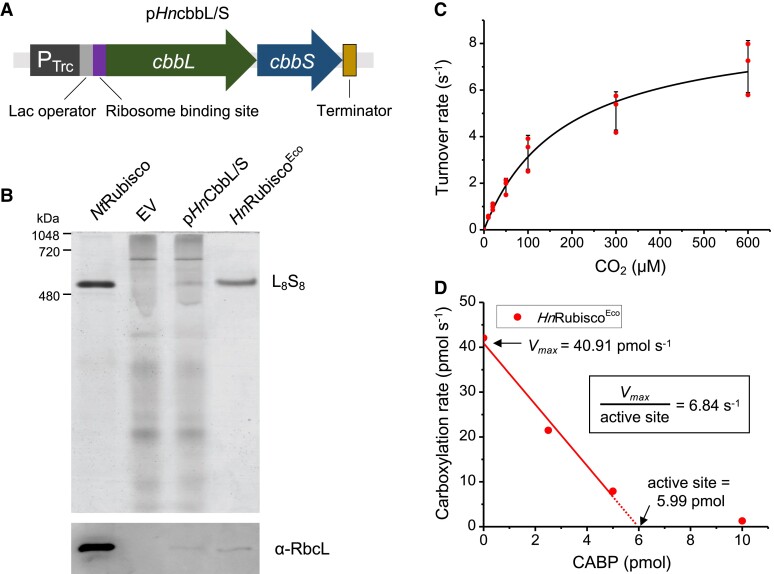
Heterologous assembly of H*n*Rubisco does not require extra chaperones in *E. coli*. A, Genetic arrangement of the CbbL/S operon in the pAM2991 vector for *E. coli* expression. B, Native-PAGE (top) and immunoblot (bottom) analysis indicate the formation of CbbL_8_S_8_ complexes. From left to right: Rubisco CbbL_8_S_8_ complexes purified from WT tobacco leaves, empty vector (EV), total soluble protein of p*Hn*CbbL/S, and *Hn*Ruibsco^Eco^ purified from p*Hn*CbbL/S. C, Carbon fixation activity of *Hn*Rubisco^Eco^ purified from p*Hn*CbbL/S at different CO_2_ concentrations, fitted with the Michaelis–Menten equation. The *k_cat_^C^* and *K_C_* values were 8.85 ± 0.5 s^−1^ and 182.4 ± 26.9 μM, respectively. Data are presented as mean ± standard deviation (SD, *n* = 3, Table 1). D, Quantification of the Rubisco active sites as a function of CABP concentration (0, 2.5, 5, and 10 pmol) based on a previously reported procedure (Davidi et al., 2020). The inhibition of CABP is described by a linear model within a certain concentration range (*R*^2^ = 0.99). The *X*-intercept indicates the concentration of Rubisco active sites, and the *Y*-intercept gives the carboxylation rate without CABP inhibition (*V*_max_). The specific activity per active site was calculated by dividing *V*_max_ by the number of active sites. Under these conditions, *Hn*Rubisco^Eco^ catalyzes 6.84 reactions per second (Table 2).

Rubisco assembly requires chaperones in native and non-native hosts (Aigner et al., 2017; Hayer-Hartl and Hartl, 2020; Lin et al., 2020). It was shown that expression of the *H. neapolitanus* α-carboxysome operon in *E. coli* could result in the generation of functional α-carboxysomes that encapsulate functional Rubisco complexes (Bonacci et al., 2012; Chen et al., 2022), suggesting that no cognate chaperone is requiremed for the production of *H. neapolitanus* Form IA Rubisco (*Hn*Rubisco). To verify the expression and assembly of *Hn*Rubisco, we expressed a p*HncbbLS* vector containing the *cbbL* and *cbbS* genes from *H. neapolitanus* in *E. coli* under isopropyl β-D-1-thiogalactopyranoside (IPTG) induction (Figure 1A). Native-PAGE and immunoblot analysis of cell lysates showed that the expressed CbbL and CbbS subunits could assemble to form functional CbbL_8_S_8_ complexes in *E. coli* with the same molecular mass as native tobacco Rubisco complexes (∼520 kDa) (Figure 1B). ^14^CO_2_ fixation assays confirmed the carboxylation activity of recombinant *Hn*Rubisco purified from *E. coli* (*Hn*Rubisco^Eco^) (Figure 1, C and 1D). The maximum carboxylase turnover rate (*k_cat_^C^*) of the Rubisco and the Michaelis–Menten constants for CO_2_ (*K_C_*) were 8.9 ± 0.5 s^−1^ and 182.4 ± 26.9 μM (*n* = 3, Table 1), respectively, which is consistent with the reported kinetic parameters of native *Hn*Rubisco (Dou et al., 2008; Tsai et al., 2022) and cyanobacterial Form 1A and Form IB Rubisco (Long et al., 2018; Davidi et al., 2020). *Hn*Rubisco has a two-fold greater *k_cat_^C^* and an eight-fold higher *K_C_* than plant Rubisco (*k_cat_^C^* = ∼2–5 s^−1^, *K_C_* = ∼20 μM) (Whitney et al., 2011b; Flamholz et al., 2019; Davidi et al., 2020; Gunn et al., 2020; Martin-Avila et al., 2020), confirming that *Hn*Rubisco has a faster catalytic rate than plant Rubisco, although it has a lower CO_2_ affinity. Our results also indicate that the assembly of functional *Hn*Rubisco in *E. coli* does not require any cognate chaperones, which may facilitate the engineering of functional Rubisco in crop plants.

**Table 1:** Catalytic parameters of purified Rubisco from *Escherichia coli* and chloroplasts.

Parameters	*Hn*Rubisco^Eco^	*Hn*Rubisco^Tob^	Native *Nt*Rubisco
*k_cat_^C^*(s^−1^)	8.9 ± 0.5	10.0 ± 0.4	3.6 ± 0.1
*K_C_*(μM)	182.4 ± 26.9	166.1 ± 18.3	22.8 ± 2.8

*n = 3* independent biological replicates.

### Chloroplast transformation in tobacco

**Figure 2:**
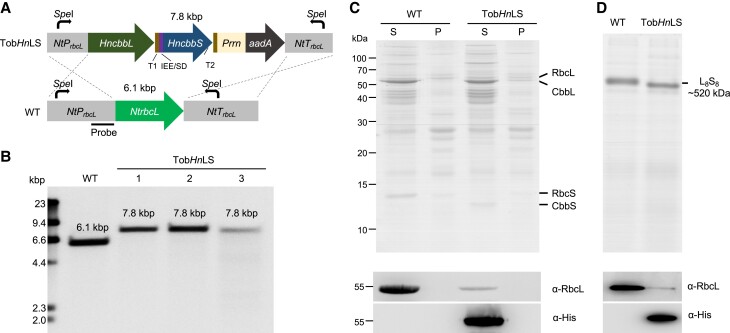
Engineering *Hn*Rubisco in tobacco. A, Gene organization of the *HncbbLS* locus in the Tob*Hn*LS construct, which was transformed into wild-type (WT) tobacco chloroplasts to replace the endogenous *NtrbcL* gene. T1, *AtTpet* D; T2, *AtTpsb* A; IEE, intercistronic expression elements; SD, Shine–Dalgarno sequence. B, DNA gel blot of total genomic DNA of WT and Tob*Hn*LS transgenic plants digested by *Spe* I using the probe indicated in A. A fragment length polymorphism was detected between the transgenic lines and WT. The shifting of the fragment length polymorphism confirmed the complete segregation of the *HncbbLS* operon into the tobacco chloroplast genome, resulting in homoplasmy. The sizes of DNA markers are indicated in kbp. C, SDS-PAGE (top) and immunoblot analysis (bottom) of total soluble proteins (S) and insoluble proteins (P) indicate the successful expression and solubility of *Nt*RbcL/*Hn*CbbL in both WT and Tob*Hn*LS transgenic plants. CbbL/RbcL are ∼50 kDa in size, according to immunoblot analysis using an anti-RbcL antibody. The analysis was performed based on equal protein loading. D, Native-PAGE (top) and immunoblot analysis (bottom) of total soluble proteins confirm that the expressed *Hn*CbbL and *Hn*CbbS form Rubisco CbbL_8_S_8_ complexes (∼520 kDa).

To express *Hn*Rubisco in tobacco (*Nt*) chloroplasts, we designed pTob*Hn*LS, a plastome transformation vector that includes the *HncbbL* and *HncbbS* operon as well as the necessary elements for gene transcription and translation in chloroplasts, including terminators, an intercistronic expression element (IEE), and Shine–Dalgarno (SD) sequences (Figure 2A). The *aadA* gene (encoding aminoglycoside (3') (9) adenylyltransferase) conferring spectinomycin resistance, driven by the *Prrn* (rRNA operon promoter), was inserted downstream of *HncbbS*. A 6X-Histidine tag was fused to the C-terminus of CbbL to facilitate differentiation of *Nt*RbcL and *Hn*CbbL in transgenic plants. We transformed the pTob*Hn*LS vector into tobacco chloroplasts via biolistic bombardment to replace the endogenous *NtrbcL* gene and express *Hn*Rubisco in the chloroplasts (Figure 2A). Positive transgenic lines were obtained after two rounds of selection and regeneration, and these transplastomic plants were grown autotrophically in soil in air supplemented with 1% (v/v) CO_2_ to flowering and seed harvesting. Two independent transplastomic lines, each with three independent plants (six different plants in total), were selected for further plant performance analysis. DNA gel blot analysis using DNA fragments specific for the 5′ UTR of *NtrbcL* as the probe showed a complete replacement of the WT fragments in transgenic lines, confirming the full integration of the *HncbbLS* operon into the tobacco chloroplast genome, resulting in homoplasmy (Figure 2B).

### Assembly of functional *Hn*Rubisco hexadecamers in tobacco chloroplasts

We conducted SDS-PAGE and immunoblot analysis of total soluble proteins from tobacco leaves (equal loading) to examine the expression of the transgenic cassettes (Figure 2C). SDS-PAGE showed that the *Hn*Rubisco large subunit *Hn*CbbL and small subunit *Hn*CbbS were expressed in transplastomic leaves (Figure 2C). As the α-RbcL antibody used in this study was not able to differentiate between *Nt*RbcL and *Hn*CbbL, immunoblot analysis using an α-6X-Histidine tag antibody confirmed the expression of *Hn*CbbL in chloroplast transformants in the soluble protein fraction, indicating that almost all the *Hn*Rubisco proteins were in the soluble form in the chloroplast transformants, as in WT plants (Figure 2C). In addition, *Hn*CbbS (∼13 kDa) but not the endogenous *Nt*RbcS subunit (∼15 kDa) could be detected by SDS-PAGE in the transformants, suggesting that free *Nt*RbcS may be rapidly degraded in the tobacco chloroplast in the absence of *Nt*RbcL (Schmidt and Mishkind, 1983). Native-PAGE and immunoblot analysis further revealed that the chloroplast-expressed *Hn*CbbL and *Hn*CbbS could form CbbL_8_S_8_ complexes (Figure 2D). We also note that the expression of *Hn*Rubisco^Tob^ in chloroplasts did not result in drastic changes in chlorophyll content in the chloroplast transformants (Table 2). Thin-section transmission electron microscopy (EM) showed no obvious protein aggregation in the transgenic chloroplasts, confirming that the expression of *Hn*Rubisco did not affect the chloroplast structure (Supplemental Figure 1). Collectively, these results demonstrate the efficient assembly of *Hn*Rubisco CbbL_8_S_8_ complexes (*Hn*Rubisco^Tob^) in transgenic tobacco chloroplasts.

**Table 2:** Biochemical and physiological properties of WT and transgenic tobacco.

Parameters		WT	Tob*Hn*LS1	Tob*Hn*LS2
Rubisco content	CABP (μmol m^−2^)	6.66 ± 0.15	2.74 ± 0.05**	2.72 ± 0.05**
	Immunoblotting (μmol m^−2^)	5.85 ± 1.17	2.95 ± 0.62**	2.70 ± 0.54**
% Rubisco sites active	Initial activities (μmol min^−1^ mg^−1^)	0.25 ± 0.02	0.40 ± 0.07** (160% of WT)	0.36 ± 0.06** (138% of WT)
	Total activities (μmol min^−1^ mg^−1^)	0.26 ± 0.03	0.45 ± 0.08** (173% of WT)	0.41 ± 0.08** (164% of WT)
	%	97.02 ± 2 .07	88.99 ± 0.41**	89.47 ± 6.94**
Soluble protein content	g m^−2^	1.80 ± 0.05	1.72 ± 0.34	1.71 ± 0.3
Chlorophyll content per unit leaf area	Chl *a* (mg m^−2^)	271.90 ± 9.42	266.90 ± 11.61	259.50 ± 6.01
	Chl *b* (mg m^−2^)	103.10 ± 2.68	106.30 ± 2.95	100.40 ± 0.84
	Total (mg m^−2^)	375.00 ± 12.10	373.20 ± 14.53	359.90 ± 6.16
Respiration	CO_2_ emission (μmol m^−2^ s^−1^)	0.76 ± 0.14	1.14 ± 0.06**	1.35 ± 0.24**
Stomatal conductance (*gs*)	mol m^−2^ s^−1^	0.098 ± 0.015	0.092 ± 0.007	0.098 ± 0.004

Data are presented as mean ± standard deviation (SD, *n* = 3).

**P < 0.01 (compared with WT), one-way ANOVA test.

**Figure 3:**
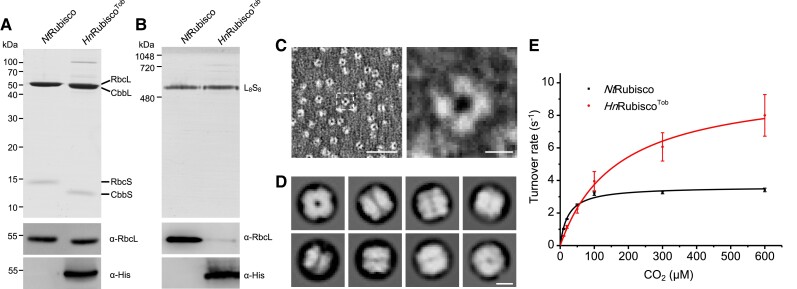
Characterization of Rubisco isolated from the leaves of wild-type (*Nt*Rubisco) and transgenic plants (*Hn*Rubisco^Tob^). A, SDS-PAGE (top) and immunoblot analysis using α-RbcL and α-6X-Histidine tag antibodies (bottom) of purified Rubisco examining demonstrating the assembly of CbbL and CbbS. No RbcS was detected in the isolated *Hn*Rubisco^Tob^, indicating that *Nt*RbcS and *Hn*CbbLS are structurally incompatible and cannot form a hybrid Rubisco complex. B, Native-PAGE (top) and immunoblot analysis (bottom) confirm that purified *Hn*Rubisco^Tob^ is in the CbbL_8_S_8_ form. C, Negative-stain EM of purified *Hn*Rubisco^Tob^ from the leaves of transgenic plants. *Hn*Rubisco^Tob^ shows a typical “dot-ring” Rubisco structure, with an average diameter of 10.7 ± 0.7 nm (*n* = 92). Scale bar: 50 nm (left), 5 nm (right). D, Selected reference-free 2D class averages of chloroplast-expressed *Hn*Rubisco^Tob^ from cryo-EM images in RELION. Scale bar: 5 nm. E, Rubisco activity assays as a function of CO_2_ concentration reveal a faster catalytic velocity in *Hn*Rubisco^Tob^ than in *Nt*Rubisco. The kinetic parameters of *Nt*Rubisco and *Hn*Rubisco^Tob^ were as follows: *k_cat_^C^* and *K_C_* of *Nt*Rubisco were 3.62 ± 0.1 s^−1^ and 22.8 ± 2.8 μM, respectively, and *k_cat_^C^* and *K_C_* of *Hn*Rubisco^Tob^ were 10.0 ± 0.4 s^−1^ and 166.1 ± 18.3 μM, respectively (*n* = 3, Table 1). Data were fitted with the Michaelis–Menten equation and are presented as mean ± SD of three independent assays.

To compare the assembly and catalytic properties of *Hn*Rubisco^Tob^ and native *Nt*Rubisco in chloroplasts, we purified *Hn*Rubisco^Tob^ and *Nt*Rubisco from transgenic and WT tobacco leaves, respectively, using rate zonal centrifugation and anion-exchange chromatography (Carmo-Silva et al., 2011). SDS-PAGE and immunoblot analysis confirmed that the tobacco-expressed *Hn*Rubisco^Tob^ was composed of CbbL and CbbS; *Hn*CbbL has a similar molecule mass to *Nt*RbcL (∼50 kDa), while *Hn*CbbS (∼13 kDa) is smaller than *Nt*RbcS (∼15 kDa) (Figure 3A). The *rbcL* gene encoding tobacco RbcL is located in the chloroplast genome, whereas several *rbcS* copies are located in the nuclear genome (Bracher et al., 2017). Our results indicate that *Nt*RbcS cannot assemble with the exogenous *Hn*CbbL subunit to form a hybrid Rubisco complex, which is consistent with the results of analysis of the total soluble protein extract (Figure 2, C and 3A), demonstrating the assembly incompatibilities between tobacco RbcS and *Hn*Rubisco L_8_S_8_ holoenzyme in chloroplasts. Native-PAGE further indicated that *Hn*Rubisco^Tob^ formed the canonical CbbL_8_S_8_ complex of ∼520 kDa, a similar size to that of native *Nt*Rubisco (Figure 3B). Negative-stain EM and cryo-EM of the isolated *Hn*Rubisco^Tob^ showed a typical ring-shaped structure of Rubisco, with 4-fold symmetry and a diameter of 10.7 ± 0.7 nm (*n* = 92) (Figure 3, C and 3D), consistent with the atomic structures of Rubisco L_8_S_8_ complexes (Huang et al., 2020; Oltrogge et al., 2020).

We measured the activities of purified *Hn*Rubisco^Tob^ and *Nt*Rubisco in ^14^CO_2_ fixation assays as a function of CO_2_ concentration. The *k_cat_^C^* and *K_C_* of isolated *Hn*Rubisco^Tob^ were 10.0 ± 0.4 s^−1^ and 166.1 ± 18.3 μM (*n* = 3), respectively (Figure 3E, Table 1), which are of the same magnitude as those of *E. coli*-expressed *Hn*Rubisco^Eco^ (Figure 1C, Table 1) (Tsai et al., 2022). By contrast, native *Nt*Rubisco exhibited a ∼2-fold lower *k_cat_^C^* (3.62 ± 0.1 s^−1^, *n* = 3) and a ∼7-fold lower *K_C_* (22.8 ± 2.8 μM, *n* = 3) (Figure 3D, Table 1), in line with previously reported values (Davidi et al., 2020). Our activity assays revealed that *Hn*Rubisco^Tob^ has a two-fold faster carboxylation rate than the native *Nt*Rubisco enzyme and the red-type Form 1C Rubisco previously expressed in chloroplasts (Gunn et al., 2020); the carboxylation rate of *Hn*Rubisco^Tob^ is similar to those of cyanobacterial Rubisco (Long et al., 2018; Davidi et al., 2020).

### *Hn*Rubisco production and function in transgenic chloroplasts

Our SDS-PAGE analysis of total soluble proteins suggested that the Rubisco content was reduced in transgenic chloroplasts compared with the WT (Figure 2C). To test this, we quantified the Rubisco content by both examining the regression of Rubisco activity versus the concentration of carboxyarabinitol-1,5-bisphosphate (CABP) and by immunoblot analysis using an α-RbcL antibody and purified *Nt*Rubisco as reference (Figure 1D, Supplemental Figure 2). We found that the *Hn*Rubisco^Tob^ content in the Tob*Hn*LS transgenic chloroplasts was ∼40% the level of *Nt*Rubisco present in WT tobacco chloroplasts (Table 2); this value is similar to the content of red-type Form 1C Rubisco expressed in tobacco chloroplasts but much greater than the yields (∼10%) of cyanobacterial Rubisco produced in tobacco chloroplasts (Lin et al., 2014; Occhialini et al., 2016; Long et al., 2018; Gunn et al., 2020).

Nevertheless, total Rubisco activity is affected by not only the Rubisco content but also the number of activated sites. This activation is dependent on the carbamylation of Lys201 and Mg^2+^ to form an active state and is usually inhibited by the binding of substrates (such as ribulose 1,5-bisphosphate, RuBP) and decarbamylation of Lys201 (Andersson, 2008; Sharwood et al., 2016a). Although the amount of Rubisco was reduced, activity assays in the presence of 50 mM NaH^14^CO_3_ showed that ∼89% of the Rubisco catalytic sites were activated in transgenic chloroplasts, slightly lower than that determined for the WT (∼97%) (Table 2). In addition, *Hn*Rubisco^Tob^ had ∼2-fold higher *k_cat_^C^* than native *Nt*Rubisco (Figure 3D). These features allowed the transgenic chloroplasts to exhibit over 60% higher total carboxylation activity than the WT (Table 2). On the other hand, this may also have resulted in the reduced Rubisco content in the chloroplast transformants relative to the WT.

### Bacterial Form-1A Rubisco-driven plant growth

**Figure 4:**
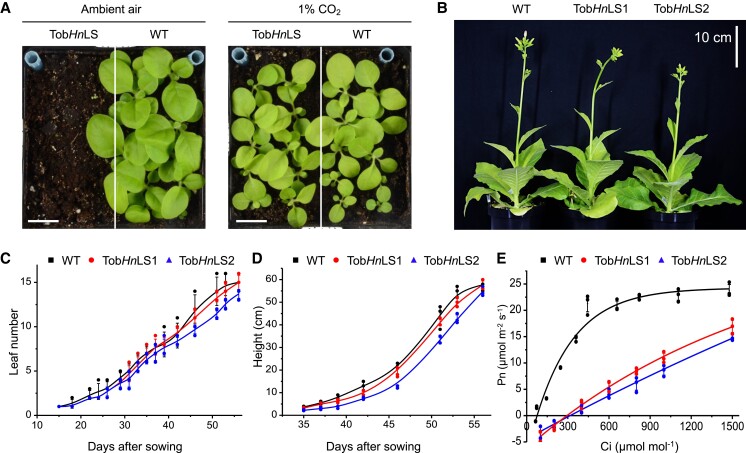
*Hn*Rubisco supports autotrophic growth of tobacco plants in air with 1% CO_2_. A, Phenotypes of the transgenic plants and WT grown at 25°C in air with or without 1% (v/v) CO_2_ on the 33rd day after sowing. The germinated seeds of WT and transgenic plants were sown in the same pot (12 cm × 12 cm) and grown in either ambient air or 1% CO_2_. With 1% CO_2_, the transgenic seeds germinated and grew as well as WT. In ambient air, however, the transgenic seeds stopped growing after germination and had completely died 33 days after sowing. See also Supplemental Figure 3. Scale bar: 2 cm. B, *Hn*Rubisco-supported growth of Tob*Hn*LS tobacco in air supplemented with 1% CO_2_ at 53 days after sowing, compared with WT. C–E, leaf number (C), plant height (D) and leaf gas-exchange measurements (E) of WT and Tob*Hn*LS transgenic plants grown in air with 1% CO_2_. Leaf gas-exchange analysis of net CO_2_ assimilation rates (Pn) as a function of intercellular CO_2_ pressure (Ci) at 25°C and 1,200 μmol photons·m^−2^·s^−1^ light density. The measurements were conducted at 42 days after sowing. Data are presented as mean ± SD of three independent transgenic lines.

The high *Kc* of *Hn*Rubisco may imply that the growth of the transgenic plants requires a high concentration of CO_2_. Indeed, seeds of the Tob*Hn*LS transgenic lines could be germinated in ambient air (∼400 ppm CO_2_), but the transgenic plants were not able to grow to maturity at the ambient CO_2_ level and were completely dead 33 days after sowing (Figure 4A, Supplemental Figure 3). Consistent with previous findings (Gunn et al., 2020), WT plants showed better growth in ambient air than in higher CO_2_ conditions (Figure 4A). Nevertheless, the transgenic plants showed essentially the same growth rate as the WT in air supplemented with 1% CO_2_, presumably due to the faster carboxylation rate of *Hn*Rubisco at high CO_2_ conditions compared with *Nt*Rubisco, while relatively poor activity was observed at low CO_2_ conditions (Figure 3D and 4A–4D). In addition, leaf photosynthetic CO_2_ response curves on the same leaves showed that the CO_2_ compensation point was ∼300 ppm (Figure 4E), which is similar to that of chloroplast transformants expressing red-type Form 1C Rubisco and slightly lower than that of transplastomic lines expressing a cyanobacterial Form 1A Rubisco (Long et al., 2018; Gunn et al., 2020).

Gas-exchange experiments revealed that the net photosynthetic rate (Pn) of the transgenic line was lower than that of the WT below 1,500 ppm CO_2_ (Figure 4E). The net CO_2_ assimilation rate of the transgenic lines was nearly 1.6 μmol m^−2^ s^−1^ in ambient CO_2_ conditions. Meanwhile, CO_2_ emission via respiration of the transgenic lines (1.14 ± 0.06 μmol m^−2^ s^−1^, *n* = 3) was slightly higher than that of the WT (0.76 ± 0.14 μmol m^−2^ s^−1^, *n* = 3) under 400 ppm CO_2_ (Table 2). Moreover, there was no marked difference in stomatal conductance (*gs*) between WT (0.098 ± 0.015 mol m^−2^ s^−1^, *n* = 3) and the transgenic line (0.092 ± 0.007 mol m^−2^ s^−1^, *n* = 3) (Table 2). Together, these results indicate that the low net rate of CO_2_ assimilation in ambient air was insufficient to support autotrophic growth. Nevertheless, the transgenic plants showed essentially the same growth rate as WT plants in 1% CO_2_, confirming the catalytic activities of the heterologously engineered *Hn*Rubisco in tobacco chloroplasts.

Our finding regarding the *Hn*Rubisco^Tob^ content (∼41% of WT) and activation states (∼89% activated catalytic sites in transgenic plants) suggest that *Hn*Rubisco^Tob^ activation in the transgenic plants is not the limiting factor in photosynthesis under elevated CO_2_ conditions. Moreover, a nearly linear relationship between Pn and the CO_2_ concentration was observed for the transgenic plants when the CO_2_ concentration was lower than 1,500 ppm (Figure 4E). The growth performance and Pn data suggest that the CO_2_ levels in chloroplasts were insufficient for the carboxylation of the fast *Hn*Rubisco^Tob^, given that the transgenic plants were grown in 1% CO_2_ and gas exchange was carried out at a high concentration of CO_2_ (up to 1,500 ppm). These results also suggest that the inorganic CO_2_ concentration in the stroma of chloroplasts may be an important factor for efficient carbon fixation, highlighting the need to increase HCO_3_^–^ diffusion and accumulation in chloroplasts by introducing bicarbonate transporters to the chloroplast envelope, along with engineering the fast *Hn*Rubisco, to enhance plant photosynthesis and growth (Price et al., 2011; Price et al., 2013).

## Discussion

Engineering Rubisco with a high carboxylation rate into plant chloroplasts represents a promising approach to improving crop performance and productivity (Zhu et al., 2010; Long et al., 2018). Here, we used model bacterial and plant systems to test the assembly and function of a Rubisco variant from the chemoautotrophic bacterium *H. neapolitanus*. We demonstrated the efficient production of the *H. neapolitanus* Form 1A Rubisco CbbL_8_S_8_ complex, with a high carboxylation rate in *E. coli* and tobacco chloroplasts and no requirement for cognate assembly chaperones. Our results show that engineering *Hn*Rubisco into chloroplasts to replace the endogenous tobacco Rubisco allowed the transplastomic tobacco lines to grow at essentially the same rate as WT plants in air supplemented with 1% CO_2_. This study provides insight into Rubisco assembly and represents a step toward installing fast, highly active Rubisco as well as CCM pathways into chloroplasts to enhance crop photosynthesis and yield.

Exogenous Form 1 Rubisco enzymes with high catalytic rates have been promising targets to replace tobacco Rubisco (Lin et al., 2014; Occhialini et al., 2016; Long et al., 2018; Gunn et al., 2020; Orr et al., 2020). While several Form 1 Rubisco variants can be expressed and assembled in *E. coli* (Davidi et al., 2020), the protein components of Form 1 Rubisco complexes are prone to aggregation and require highly specialized chaperonins/chaperones for proper folding and assembly to form the final L_8_S_8_ holoenzymes (Whitney et al., 2001; Wilson and Hayer-Hartl, 2018). The biogenesis of plant Form 1B Rubisco requires several auxiliary chaperone/chaperonin components when expressed in *E. coli* (Aigner et al., 2017). Likewise, the cognate RUBISCO ACCUMULATION FACTOR 1 (*At*RAF1) specific for *Arabidopsis* Rubisco large subunits was needed to increase the assembly efficiency of recombinant Rubisco in tobacco chloroplasts (Whitney et al., 2015). By contrast, carboxysomal Rubisco from some cyanobacterial species could be assembled in *E. coli* and tobacco chloroplasts without extra chaperones (albeit generally at low efficiency) (Gatenby et al., 1985; Lin et al., 2014; Occhialini et al., 2016; Long et al., 2018), although chaperones (such as Raf1 and RbcX) play roles in mediating and promoting the assembly of cyanobacterial Form 1B Rubisco and carboxysome formation (Huang et al., 2019; Huang et al., 2020). By contrast, Rubisco from some cyanobacterial species, such as *Thermosynechococcus elongatus* BP1, could be assembled in *E. coli* but not in tobacco chloroplasts in the absence of ancillary components (Wilson et al., 2018). Our results show that a high assembly efficiency of *Hn*Rubisco could be achieved by expressing CbbL and CbbS in *E. coli* and tobacco chloroplasts without its assembly chaperones. Almost all the expressed Rubisco large and small subunits were correctly assembled to form functional CbbL_8_S_8_ complexes, suggesting that the protein folding systems [such as GroEL/ES (Georgescauld et al., 2014)] and chaperones existing in these non-native hosts can facilitate the folding and assembly of *Hn*CbbL and *Hn*CbbS to form a functional Rubisco (Figures 2 and 3).

Despite numerous attempts to express non-native Rubisco with a higher catalytic rate in plant chloroplasts, the growth of the reported transgenic lines was shown to be slow, even under high CO_2_ conditions (Lin et al., 2014; Occhialini et al., 2016; Long et al., 2018; Gunn et al., 2020; Orr et al., 2020). In the current study, the carboxylation rate (∼10 s^−1^) of *Hn*Rubisco produced in chloroplasts and *E. coli* was much higher than those of plant Rubisco (∼2–5 s^−1^) and red-type Form 1C Rubisco (3.9 s^−1^) and was comparable to the fast cyanobacterial Form 1A (9.8 s^−1^) and Form 1B Rubisco (∼9 to 12 s^−1^) (Lin et al., 2014; Occhialini et al., 2016; Long et al., 2018; Gunn et al., 2020; Matsumura et al., 2020; Orr et al., 2020). *Hn*Rubisco^Tob^ has a slightly lower ratio of activated sites that native Rubisco but a much higher ratio of activated sites than the engineered Rubisco reported previously (Gunn et al., 2020), pointing to the high efficiency of *Hn*Rubisco assembly and activation in non-native hosts. The total Rubisco carboxylation activities in the transplastomic plants were ∼160% of that in WT tobacco, probably due to the high catalytic rate and high ratio of activated *Hn*Rubisco, and were at a level that could support autotrophic growth at a similar rate to WT plants under 1% CO_2_ (Table 2, Figure 4). Collectively, our results demonstrate that *Hn*Rubisco holds promise for producing high yield, fast and active Rubisco via crop engineering.

The fast *Hn*Rubisco has evolved to have a relatively poor affinity for CO_2_. To maximize Rubisco carboxylation, *Hn*Rubisco assemblies are encapsulated together with carbonic anhydrase within the carboxysome protein shell, which is semi-permeable to catalytic substrates and products (Faulkner et al., 2020). The intrinsic features of *Hn*Rubisco highlight the necessity of Rubisco engineering by directed evolution (Zhou and Whitney, 2019), as well as introducing functional carboxysomes and CCMs into chloroplasts (Rae et al., 2017; Hennacy and Jonikas, 2020; Liu, 2022), to further boost CO_2_ assimilation of fast *Hn*Rubisco in the future. In addition, recent studies have shown that CbbO and CbbQ function as cognate *Hn*Rubisco activases to restore carboxylation by removing inhibitors from the Rubisco catalytic sites (Chen et al., 2022; Tsai et al., 2022). Co-expressing CbbQO with *Hn*Rubisco in chloroplasts may lead to enhanced CO_2_ assimilation. We also showed that the transgenic chloroplasts produced ∼41% of the Rubisco content of WT tobacco. As the endogenous encoding sequence of *NtrbcL* was replaced by the *HncbbLS* operon from the start codon without codon optimization, further modifications to improve *Hn*Rubisco production in chloroplasts and the growth of transplastomic plants in ambient air may include optimization of the IEE and the gene sequences of *HncbbL* and *HncbbS* as well as modulating the regulatory sequences to increase transcript abundance and mRNA stability (Kuroda and Maliga, 2001; Gunn et al., 2020).

## Materials and methods

### Vector construction, chloroplast transformation, and DNA gel blotting

The *cbbLS* operon was amplified from pHnCBS1D (Bonacci et al., 2012) by PCR and assembled into pAM2991 (spectinomycin-resistance gene was changed to the kanamycin-resistance gene) by Gibson assembly (NEB).

The upstream and downstream sequences of endogenous *rbcL* were amplified from tobacco genomic DNA as the homologous recombination sites. In addition, the selection gene (*aadA*) was amplified from pZF75 (Zhou et al., 2007). These three amplicons were assembled into pEASY®-Blunt Zero (TransGen Biotech, Beijing, China) to generate the chloroplast transformation vector (pTPTR, plasmid for Tobacco Plastid Transformation of RbcL). The *cbbL* sequence was amplified from pHnCBS1D (Bonacci et al., 2012) by PCR, and the coding sequence of 6X-His tag was fused to the 3′ end of the coding sequence in the synthetic primer. IEEs, the SD sequence, *cbbS*, and terminators were designed and synthesized by GenScript (https://www.genscript.com/, Nanjing, China). The cassettes were assembled into pTPTR by Gibson assembly. The primers used in this study are listed in Supplemental Table 1.

Approximately 20 μg plasmid DNA was coated with gold particles and introduced into tobacco leaves by bombardment as previously described (Zhou et al., 2007). The bombarded leaves were cut into 5 mm × 5 mm pieces and cultured on RMOP medium [Murashige and Skoog (MS) medium with 3% (w/v) sucrose, 500 mg L^−1^ spectinomycin, 1 mg L^−1^ 6-benzylaminopurine, 0.1 mg L^−1^ naphthaleneacetic acid, 1 mg L^−1^ thiamine-HCl, 100 mg L^−1^ myo-inositol, 0.3% (w/v) Phytagel, pH 5.8]. The positive shoots were cut into small pieces for a second round of regeneration on the same RMOP medium. The shoots from the second-round selection were transferred into rooting medium [1/2 MS medium with 3% (w/v) sucrose, 500 mg L^−1^ spectinomycin] and transplanted into soil in pots for growth in a plant growth chamber (Sanyo, Japan) containing 1% (v/v) CO_2_ (25/20°C day/night, 12/12 h light/dark, and ∼450 μmol photons m^−2^ s^−1^). Three independent transplastomic plants were selected for further analysis.

Genomic DNA was extracted from the leaves as previously described (Chen et al., 2017). Approximately, 3 μg genomic DNA was digested with SpeI and separated by 1% (w/v) agarose gel electrophoresis. The DNA was transferred to a membrane (Amersham, http://www.amershambiosciences.com/) by the capillary method (Southern, 1975). DNA gel blotting was carried out following Roche's manual (https://www.roche.com/) and imaged on the ImageQuant™ LAS 4000 system (GE Healthcare, United States).

### Protein isolation and characterization

Protein extraction buffer [50 mM EPPS, 10 mM MgCl_2_, 1% (w/v) polyvinylpolypyrrolidone (PVPP), 5 mM dithiothreitol (DTT), and 1% (v/v) protease inhibitor, pH 8.0] was balanced with N_2_ gas for 30 min before being used in order to remove CO_2_. Leaf samples (2 cm^2^) were weighed and thoroughly homogenized in 1 mL pre-cooled extraction buffer. The homogenate was centrifuged at 12,000 g at 4°C for 5 min to remove cellular debris. The supernatant was analyzed as the soluble protein fraction. The pellets (insoluble proteins) were washed three times with extraction buffer without PVPP and resuspended in 300 μl extraction buffer. Both samples were mixed with 100 μl 4× SDS Sample Buffer and denatured at 100°C for 10 min. Equal volumes of soluble and insoluble samples were loaded onto an SDS-PAGE gel for immunoblotting to quantify the solubility of Rubisco.

Native and engineered Rubisco were purified by the ammonium sulfate method from tobacco leaves as described previously (Carmo-Silva et al., 2011). For the expression and purification of *Hn*Rubisco^Eco^ in *E. coli* strain BL21(DE3), each positive clone was grown in 20 mL LB (lysogeny broth) culture with 50 μg mL^−1^ kanamycin at 37°C overnight. The culture was diluted into 800-mL medium in a 2-L flask and cultured at 37°C for 2–3 h. IPTG was added to a final concentration of 50 μM to begin protein induction when the OD_600_ reached 0.6. After overnight induction, the cells were collected at 10,000 g for 10 min and washed with 20 mL basic extraction buffer [50 mM Tris–HCl, 20 mM MgCl_2_, 20 mM NaHCO_3_, and 0.2 mM EDTA (ethylenediamine tetraacetic acid), pH 7.6]. The cells were resuspended in 20 mL basic extraction buffer containing 10% (v/v) CelLytic™ B cell lysis reagent (Sigma–Aldrich, USA) and 1% (v/v) Protease Inhibitor Cocktail (Melford, UK) and broken by sonication. After centrifugation at 10,000 *g* for 10 min to remove cellular debris at 4°C, the supernatant was used for Rubisco purification following the same protocol used for plant Rubisco purification (Carmo-Silva et al., 2011).

After quantification using the Bradford method (Bradford, 1976), protein samples were denatured by adding 4× SDS Sample Buffer and heating at 100°C for 10 min and were loaded onto 15% (w/v) SDS-PAGE gels. Immunoblotting analysis was carried out using the primary rabbit polyclonal anti-RbcL antibody (Agrisera, AS03 037, dilution 1:10,000), the primary mouse monoclonal anti-6X-His antibody (Promega, dilution 1:10,000), the horseradish peroxidase-conjugated goat anti-mouse secondary antibody (Promega, W4021, dilution 1:10,000), and Goat anti-Rabbit horseradish peroxidase-conjugated antibody (Agrisera AS10 1461, dilution 1:10,000) as previously described (Sun et al., 2019; Huang et al., 2020). For native-PAGE, the samples were mixed with a 4× native Sample Buffer and separated in 7% (w/v) native-PAGE gels. After 1-h incubation in SDS transfer buffer (0.1% (w/v) SDS, 25 mM Tris, 192 mM glycine, 20% (v/v) methanol, pH 8.3), protein transfer and immunoblot analysis of the native-PAGE gels were conducted as described for SDS- PAGE gels.

### Rubisco activity assays

Activity assays and quantification of the active sites of purified Rubisco were performed using a modified titration of CABP method as previously reported (Davidi et al., 2020). In detail, NaH^14^CO_3_ was added to N_2_ gas-treated Rubisco activity assay buffer (100 mM EPPS, 20 mM MgCl_2_, 50 U mL^−1^ carbonic anhydrase, pH 8.0) to prepare reaction buffer containing 0.7 to 48 mM NaH^14^CO_3_ (corresponding to 10–600 μM CO_2_). 5 µL purified Rubisco was pre-incubated reaction buffer for 5 min, and the reaction was started by adding RuBP to a concentration of 1 mM at 25°C and terminated after 5 min incubation by adding 10% (v/v) formic acid. The samples were dried on heat blocks at 100°C to remove the free NaH^14^CO_3_. The pellets were resuspended in 200 µL distilled water and mixed with 2 mL scintillation cocktail (Ultima Gold XR, Perkin–Elmer, USA). Radioactivity measurements were carried out using a scintillation counter (Tri-Carb, Perkin–Elmer, USA). Raw readings were converted to the amount of fixed^14^ C according to the standard curve. Meanwhile, 5 µL Rubisco samples were pre-incubated in reaction buffer containing 0, 10, 20, and 40 nM CABP for 15 min at 25°C for Rubisco quantification. The reaction started by adding RuBP to 1 mM and terminated after 5 min incubation by adding 10% (v/v) formic acid. The intercept with the *x*-axis represents the number of Rubisco active sites as a function of CABP concentration (in nmol; Figure 1D).

The activation status of Rubisco was analyzed using a modified method based on the NADH-coupled spectrophotometric protocol (Sharwood et al., 2016a). In detail, the supernatant was analyzed directly to obtain the initial activity. A separate 100 μL aliquot of the supernatant was treated with a final concentration of 50 mM NaHCO_3_ at 4°C for 30 min to fully activate the Rubisco sites. Rubisco activity assay buffer (100 mM EPPS, 20 mM MgCl_2_, pH 8.0) was treated with N_2_ gas for 30 min before analysis. 5 µL of sample was added to the final reaction buffer (100 mM EPPS, 20 mM MgCl_2_, 50 mM NaH^14^CO_3_, 1 mM RuBP, and 50 U mL^−1^ carbonic anhydrase, pH 8.0) to initiate the reaction at 25°C, and the reaction was terminated after 5 min incubation by adding 10% (v/v) formic acid. The remaining steps were performed as described above, and the data were used to estimate the activation status of Rubisco.

### Quantification of chlorophyll content

Chlorophyll was extracted from leaf samples (2 cm^2^) using 2 mL chlorophyll extraction buffer [ethanol, acetone, and water (4.5:4.5:1, v:v:v)] in the dark at 4°C until the leaves turned entirely white. The chlorophyll samples were examined by the spectrophotometric method using a NanoDrop Ds-11 (DeNovix, USA), and chlorophyll content was calculated based on the equations of Lichtenhaler and Wellburn (Lichtenthaler and Wellburn, 1983).

### Plant growth and gas-exchange measurements

Sterilized tobacco (*Nicotiana tabacum* cv. Petit Havana) seeds were sown on MS medium containing 3% (w/v) sucrose. For growth tracking and gas-exchange analysis, the germinated seeds were transferred to a pot containing Levington F2S Seed & Modular Compost and Vermiculite Medium (v:v = 3:1). WT and two transgenic lines were cultured individually in three biological replicates (three WT plants and six different transgenic plants in total) in an environment-controlled chamber (Sanyo, Japan) with 1% (v/v) CO_2_, 25/20°C day/night, 12/12 h light/dark, and ∼450 μmol photons m^−2^ s^−1^(LED, Wavelength: 276pcs white 3500 K, 24pcs red 660 nm). The leaf number and plant heights were recorded during the entire growth procedure. Gas exchange over the range of internal CO_2_ partial pressure (Ci, μbar) was examined at 25°C and 1,200 μmol photons m^−2^s^−1^ using the portable flow-through LI-6400 gas-exchange system (LI-COR, Nebraska, USA). In detail, fully light adapted plants were treated with different concentrations of CO_2_ (Cr, reference CO_2_ concentrations: 50; 100; 200; 300; 400; 600; 800; 1,000; 1,200; 1,500; and 2,000 ppm). Gas-exchange data were modeled and calculated as described previously (Farquhar et al., 1980; von Caemmerer, 2000). After full dark adaptation, the respiration of the plants was examined using an LI-6400 gas-exchange system (LI-COR, Nebraska, USA) at 25°C, 0 μmol photons m^−2^ s^−1^, and 400 ppm CO_2_; the result was defined as the CO_2_ emission rate.

### Negative-stain EM and cryo-EM

The structures of purified Rubisco were characterized by negative-stain EM as described previously (Faulkner et al., 2017; Sun et al., 2019). Leaf tissue (2 mm × 2 mm) was cut and fixed by fully submerging in 3% (v/v) glutaraldehyde with 1% (v/v) paraformaldehyde in 0.1 M sodium cacodylate to observe chloroplast ultrastructure. Samples are processed using a Pelco BioWave Pro laboratory microwave system. Fixation was performed by three steps of 100 W treatment for 1 min each. The fixed leaves were washed three times in 0.1 M sodium cacodylate buffer (pH 6.8), and a secondary fixative was applied [0.5% (w/v) osmium tetraoxide in 0.1 M sodium cacodylate]. The samples were incubated at 100 W for 12 min, and the leaf tissue was serially dehydrated and embedded in LR white resin. Finally, 70–80 nm ultrathin resin sections were cut and stained with 2% (w/v) uranyl acetate and lead citrate. Both leaf sections and purified carboxysomes were observed at 120 kV on a FEI Tecnai G2 Spirit BioTWIN transmission EM with a Gatan Rio 16 camera.

For cryo-EM, purified proteins were diluted to a final concentration of 1.2 mg mL^−1^ in 10 mM Tris–HCl, 10 mM MgCl_2_, 1 mM EDTA, and 20 mM NaHCO_3_ (pH 8.0). The samples were applied to a glow discharged Quantifoil R1.2/1.3 holey carbon films, with 200 mesh copper (Agar Scientific AGS143-1-100) and blotted for 1 s with force −2 in a Vitrobot Mark IV system. A total of 50 micrographs were collected at 120 kV on an FEI T12 transmission electron microscope with 1.0-s exposure time at a magnification of 67,000*x* with a TVIPS XF416 4K camera, resulting in pixel size of 2.31 Å. A defocus range of −1.5 to 2.1 μm was used. Single particles were selected automatically and processed with Relion 3.1.3. A total of 53,884 particles were used for reference-free unbiased 2D classifications.

### Statistical analysis

For multiple comparisons, the statistical analyses were performed using one-way ANOVA test in Origin 2021b (OriginLab, USA). All data points and *P*-values can be found in Supplemental Data Set 1.

### Accession numbers

Sequence data from this article can be found in the KEGG database under the following accession numbers: Hneap_0922 (*cbbL*) and Hneap_0921 (*cbbS*).

## Supplemental data

The following materials are available in the online version of this article.

**Supplemental Figure S1**. Transmission electron micrographs of leaf sections of WT and transgenic plants (Tob*Hn*LS).

**Supplemental Figure S2**. Quantification of the Rubisco content in tobacco leaves.

**Supplemental Figure S3**. Phenotypes of the transgenic plants and WT grown at 25°C in air with or without 1% (v/v) CO_2_.

**Supplemental Table S1**. Primers used in this study.

**Supplemental Data Set S1**. Statistical analyses.

## Supplementary Material

koac348_Supplementary_Data
