# Supplementary material for: Producing fast and active Rubisco in tobacco to enhance photosynthesis
Source: Plant Cell. 2022 Dec 6;35(2):795–807. doi: 10.1093/plcell/koac348 (PMC9940876; doi:10.1093/plcell/koac348)
Supplement: koac348_Supplementary_Data [file koac348_supplementary_data.zip › TPC2022RA00954DR1_Supplemental Figures 13 Table 1.pdf]

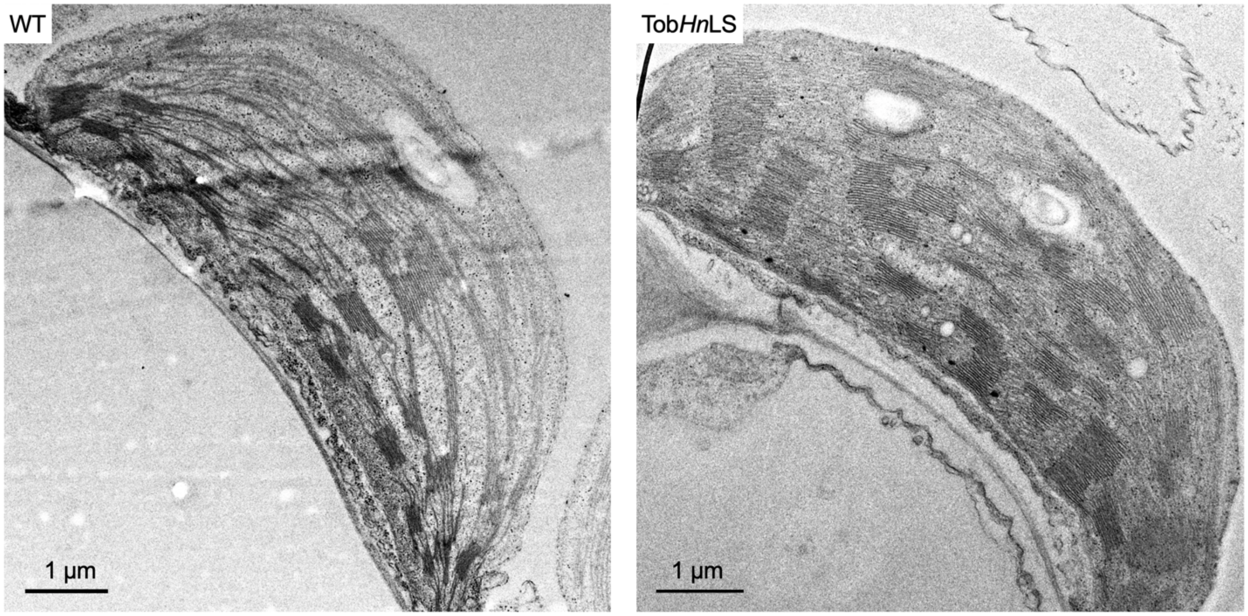

Supplemental Figure S1. Transmission electron micrographs of leaf sections of WT and transgenic plants (Tob*Hn*LS) (Supports Figure 2). No visible Rubisco aggregation or differences in thylakoids were found in Tob*Hn*LS (right) compared to WT (left), indicating the physiological distribution of *Hn*Rubisco within the chloroplasts of transgenic plants.

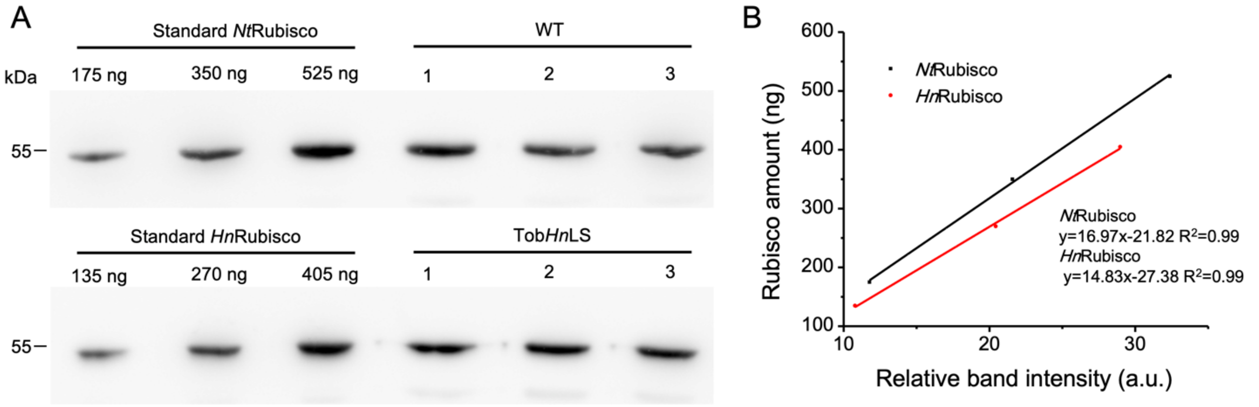

Supplemental Figure S2. Quantification of Rubisco content in tobacco leaves (Supports Figure 3). **A**, Quantification of the Rubisco content in WT and transplastomic tobacco leaves by immunoblotting using an α-RbcL antibody. The isolated Rubisco proteins from WT tobacco leaves were loaded at different concentrations as a control. See also Figure 3A. **B**, Correlation between the Rubisco content and immunoblot band density in **A**.

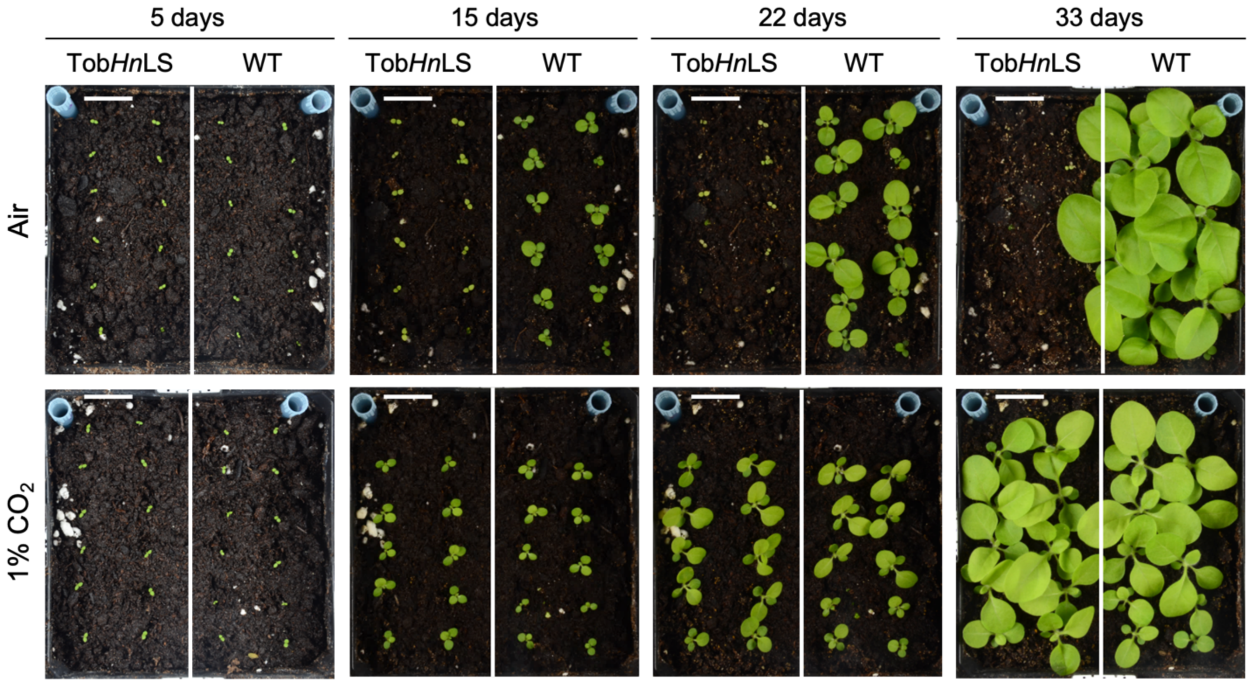

Supplemental Figure S3. Phenotypes of the transgenic plants and WT grown at 25°C in air with or without 1% (v/v) CO_2 (Supports Figure 4). The germinated seeds of WT and transgenic plants were sown in the same pot (12 cm × 12 cm) and grown in ambient air and 1% CO_2. At 1% CO_2, the transgenic seeds germinated and grew similarly to the WT. In ambient air, however, the transgenic seeds stopped growing after germination and the plants completely died 33 days after sowing (see Figure 4A). Scale bar: 2 cm.

Supplemental Table S1. Primers used in this study. Homologous sequences for Gibson assembly and His-tag coding sequences are shown in bold and underlined, respectively.

Primer	Sequence
pAM2991.CbbL-F	**cacaggaaacagaccatggaattc**atggcagttaaaaagtatagtgctggtg
pAM2991.CbbS-R	**ctgcaggtcgactctagaggatcc**cttgaataagtgctgtgcgtagag
pEASY-F	tgcagatatccatcacactggc
pEASY-R	actagtccctttagtgagggttaattc
5'UTR-F	**ggattgattctcataataataaaat**ggattgattctcataataataaaat
5'UTR-R	gtcgacaaatccctccctacaactc
aadA-F	**gggagggatttgtcgac**gagtctaattaatcgacagatcccga
aadA-R	**gctaatgtctactgtttttac**aagcttatttgccgactaccttggtg
3'UTR-F	gtaaaaacagtagacattagcagataa
3'UTR-R	**ccagtgtgatggatatctgca**gaattcttgccccctatttgcatgaaaatac
pTPTR-F	taattaatcgacagatcccgaattggg
pTPTR-R	gtcgacaaatccctccctacaac
pTPTR.CbbL-F	**gttgtagggagggatttgtcgac**atggcagttaaaaagtatagtgc
pTPTR.CbbL-R	tcagtgatggtgatggtgatgacgattttgagtgtcgagtttg
CbbS-F	**catcaccatcaccatcactga**ttcaatttattcaattgtaaaataaacgacg
CbbS-R	**cgggatctgtcgattaatta**aaagctttaaaatatgatactctataaaaatttgc
